# The Pelvic INFIX Technique for Unstable Anterior Pelvic Ring Fractures: Clinical Outcomes, Radiographic Results, and Complications

**DOI:** 10.3390/jcm15124594

**Published:** 2026-06-13

**Authors:** Vasileios Athanasiou, Michail Kroustalakis, Fotios Panagopoulos, Panagiotis Antzoulas, Vasileios Papathanidis, Vasileios Giannatos

**Affiliations:** Orthopedics Department, University Hospital of Patras, 26504 Patras, Greece; mikekroustalakis@gmail.com (M.K.); panfo97@gmail.com (F.P.); p.antzoulas@hotmail.com (P.A.); vasilispap2009@hotmail.com (V.P.); up1047758@ac.upnet.gr (V.G.)

**Keywords:** INFIX, pelvic, unstable

## Abstract

**Background:** Unstable pelvic ring injuries often require surgical stabilization to restore pelvic ring integrity. The anterior subcutaneous internal fixator, or pelvic INFIX, has emerged as an alternative to external fixation and open anterior fixation. This study evaluated the functional, radiographic, and complication-related outcomes of INFIX fixation for unstable anterior pelvic ring injuries. **Methods:** We retrospectively reviewed 21 adult patients treated with anterior pelvic INFIX for unstable anterior pelvic ring fractures, with or without posterior fixation, at a Level 1 Trauma Center between 2017 and 2024. Fractures were classified according to the AO/OTA system. Functional outcomes were assessed using the Iowa Pelvic Score and Short Form-12 questionnaire. Radiographic outcomes were evaluated according to Tornetta and Matta criteria. Complications were recorded throughout follow-up. The INFIX device was routinely removed 6 months postoperatively. **Results:** The cohort included 15 males and six females, with a mean age of 42.5 ± 11.1 years. Mean Injury Severity Score was 25.3 ± 9.6, and mean follow-up after implant removal was 31 (IQR 28–34) months. The mean Iowa Pelvic Score was 80.2 ± 7.4, indicating an overall good functional outcome. Mean SF-12 physical and mental scores were 49.2 ± 3.5 and 48.3 ± 7.9, respectively. Radiographic outcomes were excellent in eight patients, good in 11, and fair in two. Complications included postoperative hemorrhage, implant loosening, heterotopic ossification, and three cases of lateral femoral cutaneous nerve (LFCN) injury. **Conclusions:** INFIX fixation appears to be a reliable minimally invasive option for unstable anterior pelvic ring injuries, providing satisfactory mid-term functional and radiographic outcomes with an acceptable complication profile.

## 1. Introduction

Pelvic ring fractures typically result from high-energy trauma and account for roughly 2–8% of all fractures, while being associated with a high rate of morbidity and mortality. External fixation has been an established modality in their acute management, but it is not only used as interim stabilization. Stable fractures (AO/OTA 61-A) generally do not need surgical intervention [1]. However, when treating fractures rotationally or both rotationally and vertically unstable (AO/OTA 61-B, 61-C), surgical restoration of the pelvic ring’s structural integrity becomes a requisite, involving anterior or anteroposterior fixation [2]. As a result, many groups opt to keep the external fixation in vertically stable anteroposterior compression pelvic injuries and lateral compression pelvic injuries as the definite treatment modality [3,4,5]. Other surgeons suggest that external fixation may impose serious restrictions on patient mobility, especially when rolling from side to side or sitting, or when adjunct spinal surgery is needed, requiring prone position [6]. Moreover, it is associated with pin-tract infection (25–50%), loss of reduction (33%) and osteomyelitis (7%) [7]. Open reduction and internal fixation (ORIF), on the other hand, of comminuted, unstable anterior pelvic ring fractures is highly associated with extensive soft tissue damage [8]. The aforementioned could result in a slightly higher rate of surgical site infections and wound complications, as well as risks of neurovascular injury, damage to the bladder and spermatic cord, surgical site herniation, and even implant failure, related to EX-FIX or INFIX [9,10].

As a response to the aforementioned problems, a subcutaneous screw rod system, known as pelvic internal fixator (INFIX), was first described by Vaidya et al. [11]. It has emerged as an alternative to the external fixator when addressing pelvic ring fractures, showing a plethora of advantages, with expanded indications during the recent years [12,13,14]. The so-called pelvic bridge technique involves the placement of pedicle screws into the ilium on both sides, which are then linked by a subcutaneous rod, alongside additional fixation to the symphyseal region when necessary [12]. This provides an advantageous biomechanical profile due to a shorter lever arm and along with the denser bone of the supra-acetabular corridor, ensures a more stable fixation [15]. Other assets of the INFIX method include a reduced prevalence of soft tissue infections related to ORIF and better patient mobilization related to EX-FIX, thereby facilitating quicker recuperation [16]. Furthermore, its minimally invasive nature is considered to be less time-consuming and associated with decreased intraoperative blood loss [13]. Among the reported complications, lateral femoral cutaneous nerve injury (28%) and heterotopic ossification (9.4%) are reported along with rarer others [7,17].

We present a retrospective INFIX case series. Primary outcomes include the use of patient-reported outcome measures (PROMs) utilizing the Iowa Pelvic Score (IPS) and Short-Form Survey (SF-12) [18]. Imaging findings will be analyzed according to the Tornetta and Matta method and complications will be thoroughly recorded as our secondary outcomes.

## 2. Materials and Methods

We retrospectively reviewed 21 patients operated with INFIX for unstable anterior pelvic ring fractures, with or without adjunct posterior fixation, in a University Hospital, Level 1 Trauma Center from 2017 to 2024. All patients signed written informed consent before participation in the study. Ethical review and approval were waived for this study. According to Greek legislation (Law 3418/2005, Code of Medical Ethics), retrospective case series that utilize anonymized data from medical records and do not involve any new intervention or deviation from standard clinical practice do not constitute “interventional clinical research” requiring formal approval from the National Ethics Committee. Patient data confidentiality was ensured in strict compliance with the General Data Protection Regulation (GDPR) and Greek Law 4624/2019. Three patients in our database were lost in follow-up after the implant’s removal, thus were not included. All patients followed a complete radiological evaluation including anteroposterior, pelvic inlet and outlet x-rays preoperatively and postoperatively. All patients had a thorough neurological examination preoperatively and postoperatively The inclusion criteria were age above 18 and patients experiencing unstable anterior pelvic ring injury, which required stabilization. INFIX osteosynthesis is considered an urgent operation, thus contraindications considered included hemodynamically unstable patients and presence of open wounds on the surgical site [13].

Demographic data was recorded including age, sex, mechanism of injury, fracture type (according to OTA/AO classification) [2] as well as associated injuries using the Injury Severity Score [19]. Immediate postoperative complications were recorded and including extensive hemorrhage and anterior thigh numbness. All patients followed a standardized postoperative rehabilitation protocol with progressive weight-bearing. The INFIX device was removed in all patients at 6 months postoperatively. Regarding the clinical outcomes, Iowa Pelvic Score (IPS) [20] was chosen to evaluate the functional outcomes following pelvic ring injuries, on which studies indicating that an average score around 80–86 represents a “good” outcome [21,22]; the SF-12 questionnaire was utilized to assess patient’s mental and physical health postoperatively. Scores above 50 indicate better-than-average health, while scores below 50 suggest lower-than-average health [18]. The radiological and clinical outcomes were evaluated at the last patient’s follow-up at a minimum of 2 years after the implant’s removal. The radiological results were evaluated based on the maximal residual displacement measured on the three standard pelvic radiographs, according to the criteria defined by Tornetta and Matta. A residual displacement of 0–4 mm was classified as excellent, 5–10 mm as good, 10–20 mm as fair, and greater than 20 mm as poor [23].

### 2.1. Surgical Technique

**Figure 1:**
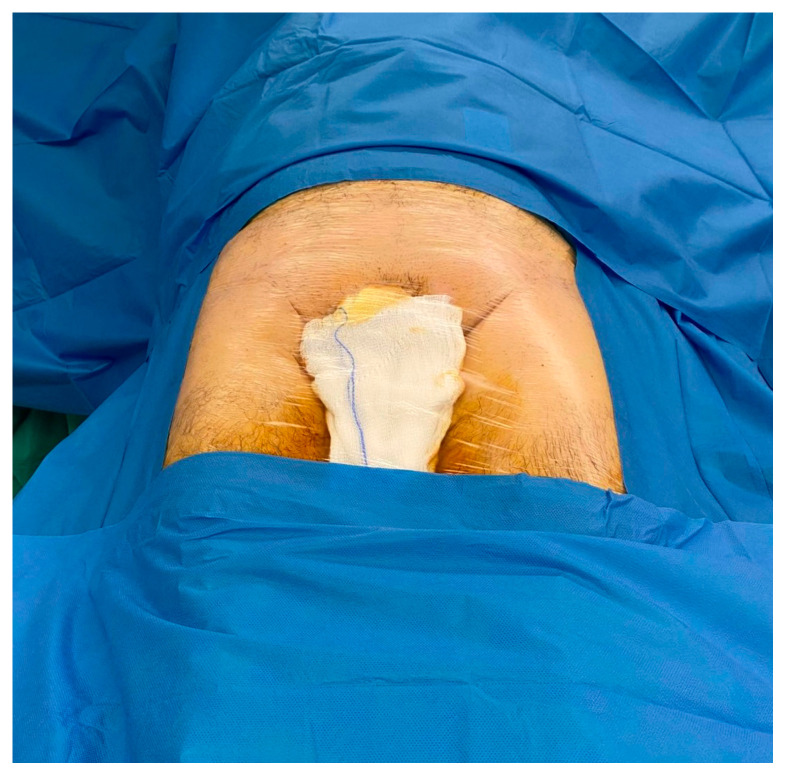
Demonstrating patient’s draping in supine position, from the umbilical level to the upper thigh.

**Figure 2:**
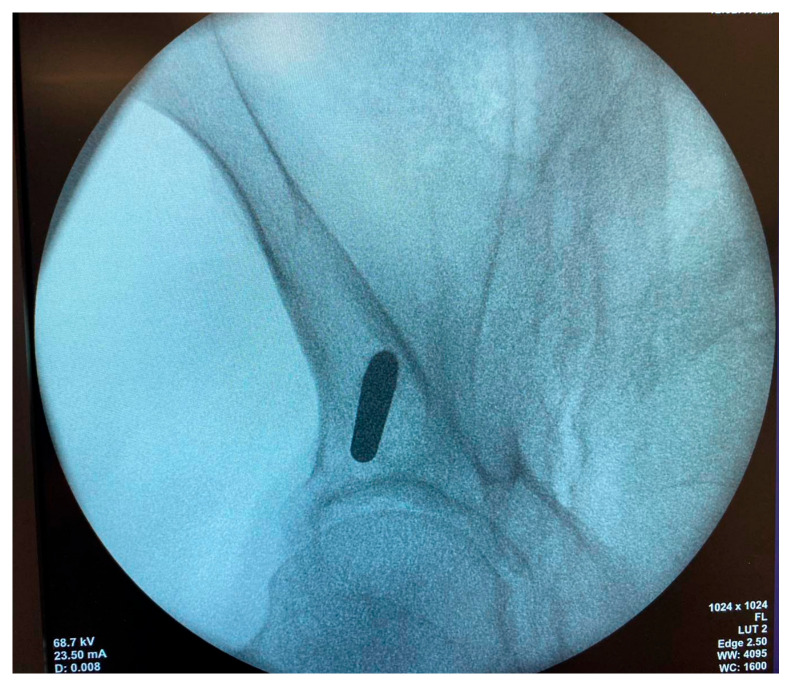
Oblique fluoroscopic view depicting the “tear-drop” target of the Schanz pin’s entry point.

**Figure 3:**
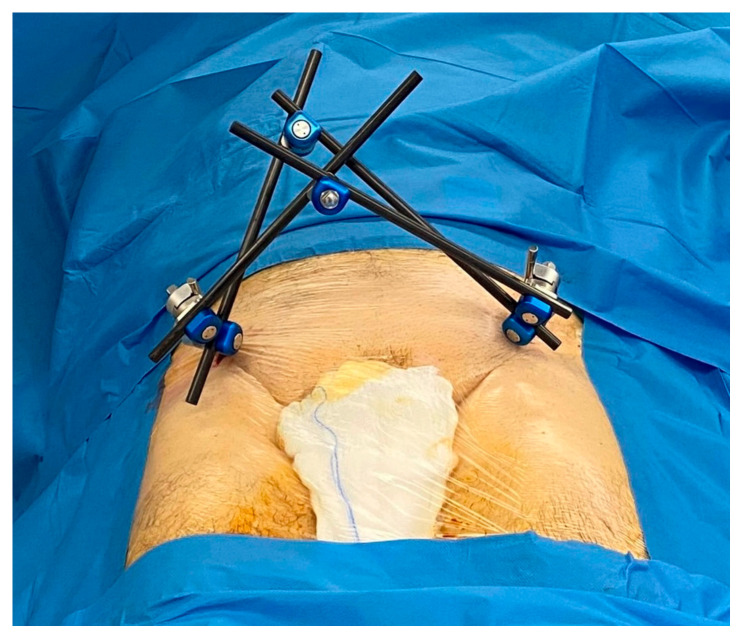
Completion of external fixation, achieving reduction in the pelvic ring and provisional stabilization.

**Figure 4:**
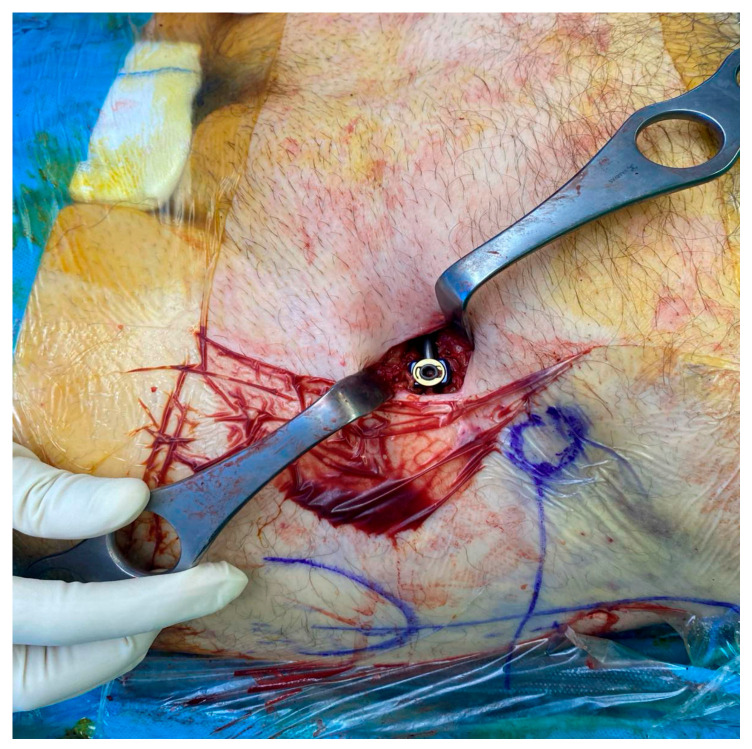
The screws positioned within the supra-acetabular corridor. Heads intentionally left prominent approximately 2–2.5 cm above the bony cortex to facilitate rod connection.

**Figure 5:**
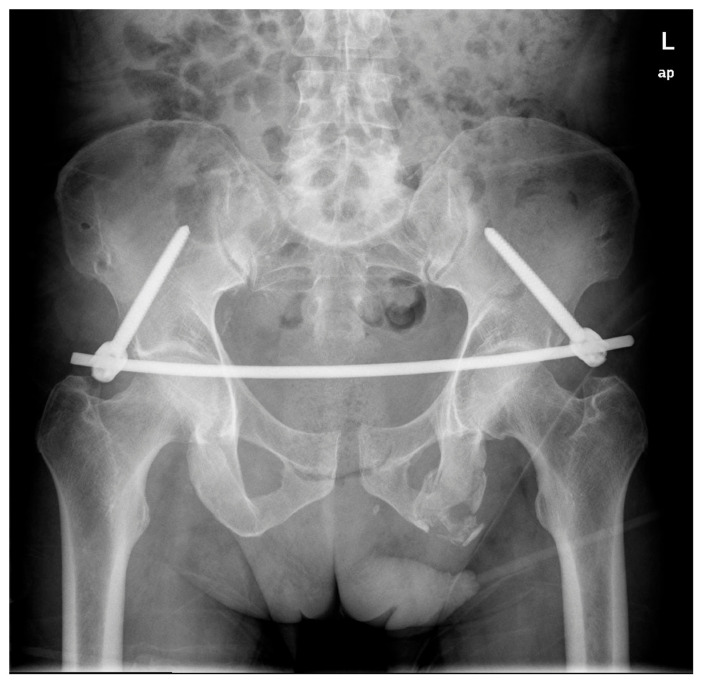
Anteroposterior pelvic view after infix implantation.

All patients were initially assessed and managed according to the principles of Advanced Trauma Life Support (ATLS). In cases where damage control surgery was indicated as the initial treatment strategy, supra-acetabular external fixation of the pelvic ring was performed, followed by placement of an anterior subcutaneous internal fixator (INFIX), with or without additional percutaneous posterior pelvic ring fixation. In hemodynamically stable patients, primary stabilization with anterior pelvic INFIX was undertaken within 24 h from the injury as a first-line modality. All procedures were performed on a radiolucent operating table under fluoroscopic guidance using a C-arm. Patients were positioned supine and prepared and draped in a sterile fashion from the umbilicus to the upper thighs (Figure 1). Fluoroscopic assessment included inlet, outlet, and oblique views. For supra-acetabular external fixation, a percutaneous needle was initially inserted and directed toward the anterior inferior iliac spine (AIIS). The correct entry point and trajectory within the supra-acetabular corridor were confirmed using oblique fluoroscopic (tear-drop) views (Figure 2). Following a small skin incision, a 6.5 mm self-tapping Schanz pin was inserted, either with or without predrilling, and advanced manually using a T-handle. Continuous fluoroscopic control was used to verify appropriate positioning and depth within the supra-acetabular corridor. During pin insertion, the hips were maintained in 40–60° of flexion to reduce tension on the lateral femoral cutaneous nerve (LFCN), thereby minimizing the risk of iatrogenic injury; as it is crucial to avoid the anatomical risk zone, at least three finger breadths medial to the anterior superior iliac spine (ASIS) were used. External fixation was then completed, achieving reduction and provisional stabilization of the pelvic ring using the remaining components of the fixation construct (Figure 3). For the INFIX technique, a 2–3 cm longitudinal or oblique (“bikini line”) incision was made over the AIIS. With the hips maintained in 40–60° of flexion to protect the LFCN, careful layer-by-layer blunt dissection was carried down to the AIIS. Gentle soft tissue traction and handling can save the LFCN from neuroapraxia at this stage. If a supra-acetabular external fixator had been previously applied, the existing Schanz pin tract was utilized for placement of pedicle screws (8.5 mm in diameter and at least 90 mm in length). The screws were positioned within the supra-acetabular corridor, with their heads intentionally left prominent approximately 2–2.5 cm above the bony cortex to facilitate rod connection (Figure 4). After secure fixation of the pedicle screws into the ilium, a connecting rod was contoured (pre-bent), cut to the appropriate length, and inserted subcutaneously along the “bikini line” corridor (Figure 5). Spinal instrumentation and materials were utilized for the pedicle screws and rods (Stryker, Portage, USA and Medacta, Castel San Pietro, Switzerland). The contouring of the rod is important in order to avoid soft tissue impingement and thus LFCN impingement within the rod and the bone. Reduction and definitive fixation of the anterior pelvic ring were then achieved. Final wound closure was performed in layers, followed by skin suturing.

### 2.2. Postoperative Protocol

**Figure 6:**
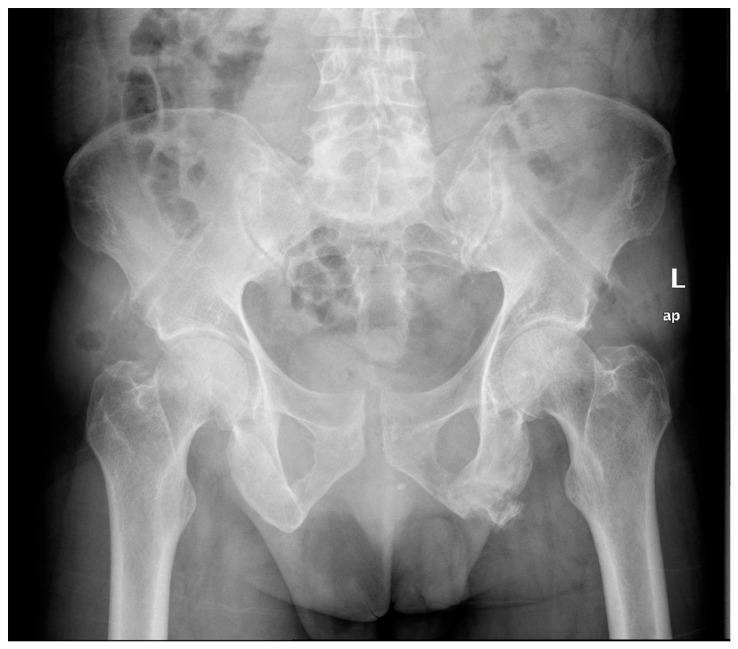
Anteroposterior pelvic view after infix explantation at 6 months.

All patients received standardized prophylactic treatment for deep vein thrombosis (DVT) with low-molecular-weight heparin (LMWH). However, no prophylactic measures were taken for heterotopic ossification prevention. All patients followed a uniform rehabilitation protocol under the guidance of the physiotherapy department of our institution. Initially the included functional exercises involved the use of the lower limbs in bed, proceeding gradually to bedside sitting with knees flexed, achieved by all our patients within the first postoperative week. Partial weight-bearing begun 4 weeks following the INFIX implantation, at which ambulation with crutches was encouraged with gradual increase in weight-bearing. Full weight-bearing and walking without crutches was not permitted until after 2 months, provided that radiological progress was evident. Removal of the implant was scheduled 6 months postoperatively both for patients with anterior ring fixation and for those with combined anterior and posterior fixation (Figure 6).

### 2.3. Statistical Analysis

The Shapiro–Wilk test was used to assess normal distribution of the data. All variables satisfied normality and were presented as mean ± standard deviation, except for last the follow-up, which was expressed as median (IQR). All statistical analysis was performed on IBM SPSS, version 24.0 (IBM, Armonk, NY, USA).

## 3. Results

The series contained 21 patients (15 males, six females) with a mean age of 42.5 ± 11.13 years, 10 of which were manual workers. The vast majority (20/21) were injured in a motor vehicle accident (MVA) except for one whose fracture was attributed to a fall from a height. The mean follow-up following the INFIX removal was 31 (IQR, 28–34) months. Fracture types were classified according to AO/OTA classification and comprising 11 vertically unstable injuries (AO/OTA type 61-C), six lateral compression injuries (AO/OTA type 61-B2), two open-book injuries (AO/OTA type 61-B1), and two bilateral “B-type’’ injuries (AO/OTA type 61-B3) [2]. Demographic data were drawn and are presented in Table 1. All the operations were performed by a single experienced trauma surgeon (V.A.). In total, 18 were operated solely for anterior pelvic ring fractures and three had combined anterior and posterior ring fractures that required stabilization.

Concerning functional outcomes, they were assessed using the Iowa Pelvic Score (IPS) during their last follow-up. The average IPS of our study group was 80.190 ± 7.42 which when graded as per Nepola et al. represents an overall “good” outcome [24]. In our series, ~33.3% of the patients (7/21) were categorized as IPS “good” outcome grade, ~19% (4/21) were graded as “excellent”, 38.1% (8/21) were graded as “fair” and 9.52% (2/21) as “poor”. The SF-12 questionnaire was deployed to appraise patient’s mental and physical health postoperatively. Regarding mental health, the average SF-12 was 48.3 ± 7.9, slightly below the expected norm in the general population. A total of 38.1% (8/21) of patients came above 50 while the rest 61.9% (13/21) fell below the general population average. As far as the patients’ physical health on the same questionnaire it averaged 48.85 ± 3.77, also close yet below the general population norm with 38.1% (8/21) sitting above and 61.9% (13/21) below [18]. Postoperative radiological results were evaluated according to the Tornetta and Matta method [23]. A typical 20% magnification was assumed for the anterior pelvic X-rays before measuring displacement with Bonesetter app (Bonesetter Solutions LLC Ann Arbor Michigan). The results were termed good or excellent in 90.48% (19/21) of the cases with 38.1% being “excellent” (8/21), 52.38% being “good” (11/21) and 9.52% being “fair” (2/21).

Every patient was able to walk without assistance and their pelvic fractures appeared healed at their last follow-up before the implant’s removal. All patients underwent the INFIX removal surgery with no major medical events. The median follow-up following the INFIX removal was 31 (IQR, 28–34). At the time of their last follow-up, patients were able to return to their occupation. In terms of complications, one patient experienced extensive hemorrhage immediately following the INFIX implantation. It was managed with spica bandaging and was resolved spontaneously, although transfusion of 1 unit of red blood cell transfusion (RBC) was required. There was an incident of implant loosening discovered during the planned operation for the INFIX removal, yet there were no significant complaints beforehand. Heterotopic ossification was experienced by one patient (male) with no reported significant functional issues up to the last follow-up. Three patients experienced anterior thigh numbness following the implant removal, attributed to lateral femoral cutaneous nerve (LFCN) injury, with one patient still experiencing it at the time of the last follow-up (neurotmesis), while numbness resolved for the other two patients at last follow-up (neuroapraxia). There were no reported episodes of deep vein thrombosis (DVT).

## 4. Discussion

Minimally invasive anterior internal pelvic fixation (INFIX) has emerged as a favorable surgical option for the management of rotationally unstable fractures of the pelvic ring. This technique incorporates the biomechanical principles of external fixation (EX-FIX) through the placement of supra-acetabular pedicle screws connected by a subcutaneous spinal instrumentation rod. Compared with traditional EX-FIX constructs, the INFIX system provides a shorter lever arm due to its subcutaneous position closer to the pelvic ring [25]. This biomechanical advantage results in improved construct stiffness and enhanced stabilization of the anterior pelvic ring [11,25].

The use of external fixation as a definitive treatment for these fractures presents several notable limitations. These include restricted patient mobility, particularly in obese individuals, as well as complications such as pin-tract infections, osteomyelitis, screw loosening, and loss of fracture reduction [26]. Several limitations for the ORIF of this approach have also been reported, including prolonged operative duration, relatively large surgical incisions, extensive periosteal stripping, and increased intraoperative blood loss [24]. Moreover, there remains a risk of postoperative infection and the potential need for revision surgery, particularly in obese patients or in individuals with a history of previous abdominal procedures [27].

Complications associated with the INFIX technique have been reported in the literature. The most commonly described complications include heterotopic ossification, injury to the lateral femoral cutaneous nerve during implant insertion or removal, infection, and femoral nerve palsy [7]. In our series, one patient experienced postoperative hemorrhage, while another demonstrated implant loosening without significant clinical symptoms. Additionally, three patients experienced injury of the lateral femoral cutaneous nerve, with one of these patients reporting persistent symptoms (neurotmesis). In a study by Steer R et al., transient irritation (neuroapraxia) of the lateral femoral cutaneous nerve was observed in 27% of patients, with symptoms resolving following implant removal. In the same series, heterotopic ossification was reported in five patients [17]. Despite precautions to protect the LFCN (hip flexion, blunt dissection), we can see from this case series, as well as the others already published, that LFCN injury is a complication associated with the procedure that one can try to minimize but cannot completely avoid, so patients should be informed about the risk preoperatively. No infections were observed in our series. This may be attributed to careful patient selection for this technique. As reported by Alencar et al., thorough preoperative assessment of the subcutaneous soft tissue envelope plays a crucial role in preventing wound dehiscence and implant exposure [26].

Loss of reduction has emerged as a controversial issue in the literature. Insufficient familiarity with the INFIX technique, particularly inadequate tightening or improper securing of the screw caps, has been reported as a potential cause of loss of reduction, occasionally necessitating revision surgery [11,28]. In our series, reduction was maintained at the final follow-up, and radiographic outcomes were assessed as good or excellent in 90.48% of cases according to the criteria described by Tornetta and Matta. Similar radiographic outcomes have been reported by Vaidya et al. and M Kuttner et al. [7,29].

Postoperative functional outcomes in our clinic were favorable as well, as all patients were able to sit, stand, and ambulate with the device in place, and this level of function was maintained until the final follow-up. The IPS of ~80.2 is accordance with the results of Steer et al. and Alencar et al. [17,26]. Although some restrictions in quality of life were observed, the outcomes of SF-12 were comparable to, or better than, those reported for other external and internal osteosynthesis techniques according to Muller et al. Our results are in line with the current literature, showing that this population fails to reach general population standards [30].

### Limitations

This study has several limitations. First, its retrospective design and relatively small sample size limit the ability to identify uncommon complications or clinically relevant subgroup differences. Second, the absence of a control group precludes direct comparison of INFIX with external fixation or open anterior internal fixation. The cohort was also heterogeneous with respect to fracture pattern and injury severity, while posterior ring fixation was applied selectively, potentially influencing both radiographic and functional outcomes. Finally, although radiographic reduction was assessed according to the Tornetta and Matta criteria, further measurements according to postoperative CT scan and reporting of measurement reliability would strengthen radiographic evaluation. Consequently, these findings should be interpreted as descriptive and hypothesis-generating. Future multicenter prospective comparative studies are required for generalization of the conclusions.

## 5. Conclusions

In this retrospective series, anterior pelvic INFIX fixation provided satisfactory mid-term functional and radiographic outcomes for patients with unstable anterior pelvic ring injuries, with an acceptable and clinically recognizable complication profile. In particular, the potential risk of lateral femoral cutaneous nerve injury should be discussed with patients preoperatively. Although the present study was not designed to establish superiority over other fixation strategies, the findings support INFIX as a useful minimally invasive option in appropriately selected patients. Further prospective comparative studies would be valuable to clarify its relative indications and outcomes compared with external fixation and open anterior fixation.

## Figures and Tables

**Table 1:** Descriptive data of the study population.

GENDER	AGE	ACCIDENT	ISS	AO/OTA Classification	LAST FOLLOW-UP M.	PIS	SF-12 P	SF-12 M	MATTA SCORE	COMPLICATIONS
M	34	MVA	21	61-B2	26	84	48,242	59,430	EXCELLENT	NONE
F	40	MVA	29	61-C1	30	82	51,569	56,880	EXCELLENT	NONE
M	60	MVA	27	61-C3	24	90	46,051	58,550	GOOD	HEMORRHAGE
M	53	MVA	14	61-C3	46	91	47,659	58,968	GOOD	HETEROTOPICOSS
M	30	MVA	34	61-B2	30	76	49,980	53,269	GOOD	NONE
M	42	MVA	18	61-B1	34	77	54,182	48,536	EXCELLENT	NONE
F	38	MVA	22	61-C1	29	82	54,356	51,898	GOOD	NEUROAPRAXIA
F	49	MVA	21	61-C1	50	73	53,983	37,792	GOOD	NONE
M	29	MVA	9	61-B2	31	91	50,237	37,166	GOOD	NONE
M	34	MVA	41	61-B3	26	79	50,665	40,079	EXCELLENT	NONE
M	61	MVA	30	61-B2	30	77	48,667	41,946	FAIR	NONE
M	51	MVA	18	61-C3	42	67	39,791	57,870	GOOD	NEUROTMESIS
F	64	FALL	9	61-B2	46	66	51,987	35,393	EXCELLENT	NONE
M	30	MVA	27	61-C1	24	82	45,767	49,214	EXCELLENT	NONE
M	43	MVA	22	61-B2	34	71	45,156	45,334	FAIR	NONE
F	33	MVA	36	61-C1	28	88	48,957	44,440	GOOD	NONE
M	30	MVA	27	61-C1	33	80	45,803	54,406	EXCELLENT	NONE
M	50	MVA	41	61-C3	50	91	49,369	49,399	GOOD	NONE
M	42	MVA	27	61-B3	24	78	52,991	37,447	EXCELLENT	NONE
F	31	MVA	18	61-B1	34	77	49,078	46,905	GOOD	NEUROAPRAXIA
M	49	MVA	41	61-C1	31	82	49,369	49,399	GOOD	NONE
	42.5 ± 11.1		25.3 ± 9.6		31 (28–34)	80.2 ± 7.4	49.2 ± 3.5	48.3 ± 7.9		

## Data Availability

The original contributions presented in this study are included in the article. Further inquiries can be directed to the corresponding author.

## Abbreviations

The following abbreviations are used in this manuscript:

ORIF	Open Reduction Internal Fixation
INFIX	Internal Fixator
PROMs	Patient-Reported Outcome Measures
IPS	Iowa Pelvic Score
SF-12	Short-Form Survey 12
ATLS	Advanced Trauma Life Support
AIIS	Anterior Inferior Iliac Spine
DVT	Deep Vein Thrombosis
LMWH	Low-Molecular-Weight Heparin
MVA	Motor Vehicle Accident
PRBCs	Packed Red Blood Cells
LFCN	Lateral Femoral Cutaneous Nerve
EX-FIX	External Fixation
